# Barcoding Sponges: An Overview Based on Comprehensive Sampling

**DOI:** 10.1371/journal.pone.0039345

**Published:** 2012-07-03

**Authors:** Sergio Vargas, Astrid Schuster, Katharina Sacher, Gabrielle Büttner, Simone Schätzle, Benjamin Läuchli, Kathryn Hall, John N. A. Hooper, Dirk Erpenbeck, Gert Wörheide

**Affiliations:** 1 Department of Earth and Environmental Sciences, Palaeontology and Geobiology, Ludwig-Maximilians-Universtität München, München, Germany; 2 Queensland Museum, South Brisbane, Queensland, Australia; 3 Eskitis Institute for Cell and Molecular Therapies, Griffith University, Brisbane, Australia; 4 GeoBio-Center^LMU^, München, Germany; 5 Bavarian State Collections of Palaeontology and Geology, München, Germany; Biodiversity Insitute of Ontario - University of Guelph, Canada

## Abstract

**Background:**

Phylum Porifera includes ∼8,500 valid species distributed world-wide in aquatic ecosystems ranging from ephemeral fresh-water bodies to coastal environments and the deep-sea. The taxonomy and systematics of sponges is complicated, and morphological identification can be both time consuming and erroneous due to phenotypic convergence and secondary losses, etc. DNA barcoding can provide sponge biologists with a simple and rapid method for the identification of samples of unknown taxonomic membership. The Sponge Barcoding Project (www.spongebarcoding.org), the first initiative to barcode a non-bilaterian metazoan phylum, aims to provide a comprehensive DNA barcode database for Phylum Porifera.

**Methodology/Principal Findings:**

∼7,400 sponge specimens have been extracted, and amplification of the standard COI barcoding fragment has been attempted for approximately 3,300 museum samples with ∼25% mean amplification success. Based on this comprehensive sampling, we present the first report on the workflow and progress of the sponge barcoding project, and discuss some common pitfalls inherent to the barcoding of sponges.

**Conclusion:**

A DNA-barcoding workflow capable of processing potentially large sponge collections has been developed and is routinely used for the Sponge Barcoding Project with success. Sponge specific problems such as the frequent co-amplification of non-target organisms have been detected and potential solutions are currently under development. The initial success of this innovative project have already demonstrated considerable refinement of sponge systematics, evaluating morphometric character importance, geographic phenotypic variability, and the utility of the standard barcoding fragment for Porifera (despite its conserved evolution within this basal metazoan phylum).

## Introduction

Sponges (Phylum Porifera), are diverse, sessile, benthic metazoans, occurring in marine, fresh-water and quasi-terrestrial ecosystems worldwide. In marine habitats, from coral reefs to abyssal plains, sponges play important roles in biogeochemical cycling [1], in the spatial structuring of the seafloor [2], and in benthic-pelagic coupling of nutrient transfer within ocean ecosystems [3]; sponges also participate in complex biotic interactions with diverse macrobiotic taxa (for a review see: [3]), and microbiological communities (e.g. [4]). According to the World Porifera Database [5], more than 8,500 species are considered valid, with most belonging to Class Demospongiae [6]. From a taxonomic and systematic point of view, Phylum Porifera is challenging because of the general paucity of characters useful for taxonomic and phylogenetic inference among sponges [7]. Furthermore, the relatively simple sponge body-plan and the ecological plasticity or evolutionary lability of the few characters available for identification make sponge taxonomy a field where uncertainty is commonplace [8], [9].

DNA barcoding has been established as an aid to increase the speed of sponge identification [10]. Although sponge mitochondrial DNA is known to evolve slow compared to other metazoans [11], DNA barcoding has been used for species identification with varying degrees of success (e.g. [12]), and to study sponge diversification patterns [13] and phylogenetic relationships [14], [15]. The Sponge Barcoding Project (www.spongebarcoding.org [16]) represents the first barcoding effort targeting non-bilaterian metazoans. The project aims to provide the most comprehensive repository of sponge barcodes, and to associate these barcodes with morphological annotations of the barcoded species. For this purpose a large number of specimens, including samples deposited in museum collections, needs to be processed (i.e. extracted, amplified and sequenced) in a time- and cost-efficient manner. Furthermore, a number of difficulties intrinsic to working with sponges need to be overcome. Barcoding sponges can be problematic due to the potentially large number of non-target macro- and microorganisms found in association with sponges [4], [17]. The DNA of these organisms can be co-extracted, and either co-amplified or preferentially amplified during PCR causing sequences to be difficult to read or to belong to non-target organisms. Moreover, for defense purposes, sponges produce potent bioactive compounds that can inhibit enzymatic reactions such as PCR [18]. Thus, a number of obstacles not usually found in other invertebrate groups needs to be tackled for successful sponge barcoding. Here, we summarize the results of an analytical pipeline established to barcode sponges and provide an overview of the current state-of-the-art on sponge barcoding that can serve other colleagues working on this challenging field.

## Results

### DNA extraction

We obtained DNA extracts of 96 families in all four classes of Porifera (Table S1). The average DNA concentration was 89±114 ng/µL (N = 156), and the mean DNA concentration of individual extraction plates ranged between 14±6 ng/µL and 191±117 ng/µL. Within-plate variation in DNA concentration values was high, and concentration differences of up to two orders of magnitude were detected within single extraction plates. Agarose gel electrophoresis revealed that the purification method was capable of recovering high-molecular weight DNA, however variability was also high among samples (N = 184) within plates. There was no apparent relationship between DNA quality, interpreted here as the presence of DNA of high molecular weight in the extracts, or DNA concentration and PCR success in the 48 samples analysed.

### PCR and sequencing success

**Figure 1:**
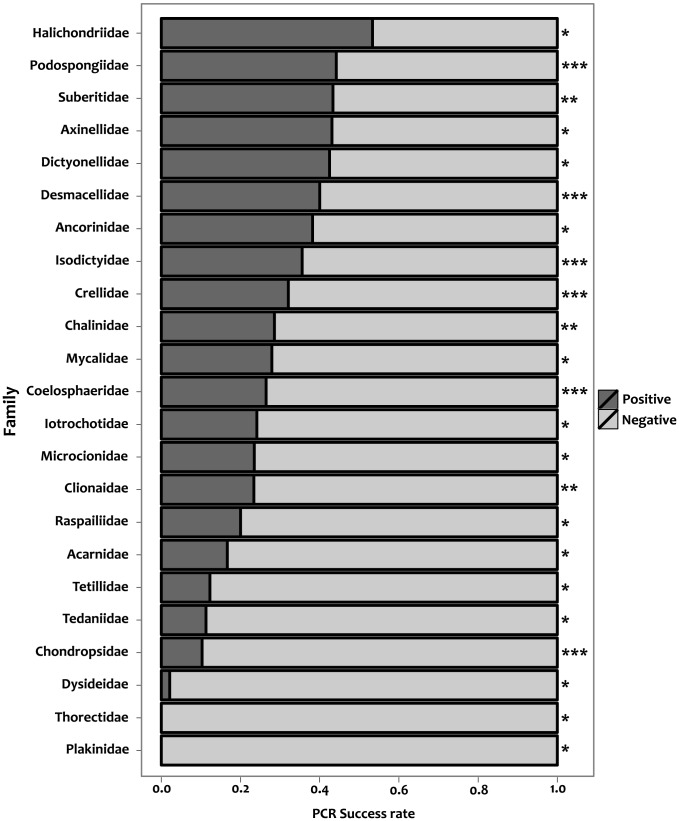
Amplification success of the standard barcoding *COI* partition per sponge family. Grey and black colours represent failed and positive reactions, respectively. Only families with more than 30 documented PCRs are included in the figure, these taxa correspond to the families analised in the generalised linear model. Asterisks on the right side correspond to the different family groups analysed: *: Acarnidae, Ancorinidae, Axinellidae, Dictyonellidae, Dysideidae, Halichondridae, Iotrochotidae, Microcionidae, Mycalidae, Plakinidae, Raspailidae, Tedaniidae, Tetillidae and Thorectidae; **: Chalinidae, Clionaidae and Suberitidae; ***: Chondropsidae, Coelosphaeridae, Crellidae, Desmacellidae, Isodictyidae and Podospongiidae.

Levels of *COI* amplification success ranged between 0% and 55% among 35 96-well plates analysed (3,360 specimens). Mean amplification success was 27±17%, which is roughly equivalent to 26 positive samples per 96-well plate. When taxonomic groups (families) were analysed (N = 73) PCR success rates covered the entire 0%–100% range, however we noted that many taxa with extreme high success rates (e.g. 100%) were represented by only few specimens. If the analysis is restricted to families with more than 30 processed specimens (N = 27; Fig. 1), PCR success levels ranged between 0% and ∼50%. Among this group, the families Dysideidae, Plakinidae, Spongiidae and Thorectidae had PCR success rates that ranged between 0% and 2% while the PCR success rates for the remaining families (N = 23) ranged between 10% and ∼50%. Among these families, PCR success rates were not independent from taxonomic membership (Table 1). PCR success rates were also affected by sample age (years post-collection) and there was a significant interaction between taxonomic membership and sample age (Table 1). These results hold for all combinations of families and age categories tested (see Methods).

**Table 1:** Generalised linear model (binomial errors, logit link) of the effect of sample taxonomic affiliation and sample age over PCR success.

Variable	Degrees of freedom	Deviance	Residual degrees of freedom	Residual deviance	Probability
	13	170.092	1485	1471.0	<0.001
Family	16	174.037	1332	1395.1	<0.001
	19	144.498	1557	1645.9	<0.001
	3	45.806	1482	1425.2	<0.001
Age category	2	21.559	1330	1373.6	<0.001
	2	36.945	1555	1609.0	<0.001
Family	39	61.062	1443	1364.1	0.0135
*	32	52.831	1298	1320.8	0.0117
Age category	38	67.156	1517	1541.8	0.0024

The reported values, are for the core family group (Acarnidae, Ancorinidae, Axinellidae, Dyctionellidae, Dysideidae, Halichondridae, Iotrochotidae, Microcionidae, Mycalidae, Plakinidae, Raspailidae, Tedaniidae, Tetillidae and Thorectidae; upper value) and for this group together with the families Chalinidae, Clionaidae and Suberitidae (middle value), and with the families Chondropsidae, Coelosphaeridae, Crellidae, Desmacellidae, Isodictyidae and Podospongiidae (lower value).

**Figure 2:**
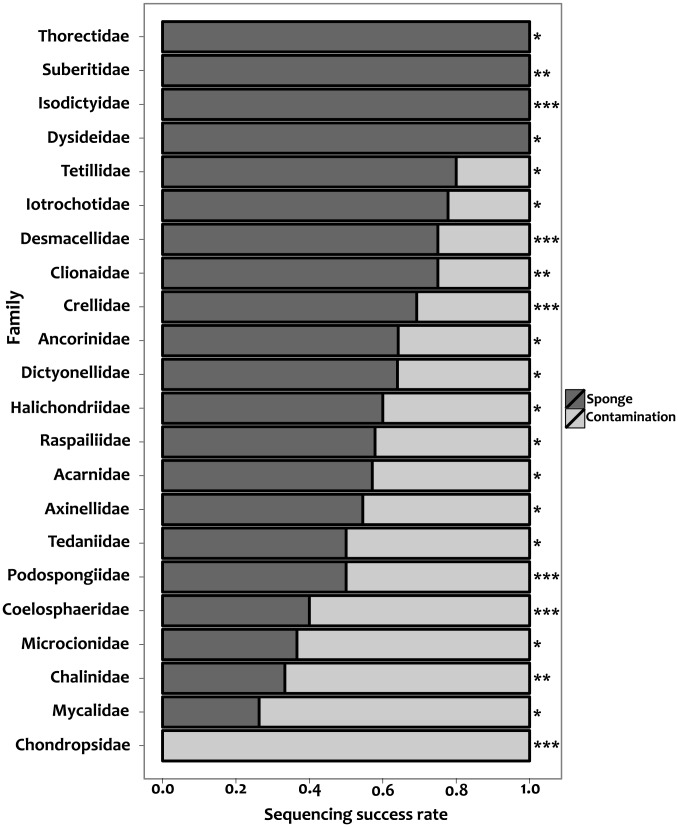
Sequencing success rates per sponge family. Grey and black colours represent sequences corresponding to non-target organisms and poriferans, respectively. The included families correspond to the families used for the analysis of PCR success, and were analised in the generalised linear model. Asterisks on the right side correspond to the different family groups analysed: *: Acarnidae, Ancorinidae, Axinellidae, Dictyonellidae, Dysideidae, Halichondridae, Iotrochotidae, Microcionidae, Mycalidae, Plakinidae, Raspailidae, Tedaniidae, Tetillidae and Thorectidae; **: Chalinidae, Clionaidae and Suberitidae; ***: Chondropsidae, Coelosphaeridae, Crellidae, Desmacellidae, Isodictyidae and Podospongiidae.

Sequencing success rates (Fig. 2), defined as the proportion of sequences of sponge origin obtained for a given sponge family or plate, were not generally affected by sample age and this variable did not interact with taxonomic assignment in any of the family groups tested (Table 2). Taxonomic membership was significant when the core family group (see Methods) was used for the analysis and when this group was used in conjunction with the families Chondropsidae, Coelosphaeridae, Crellidae, Desmacellidae, Isodyctidae and Podospongidae. The analysis of the core family group together with the families Chalinidae, Clionaidae and Suberitidae resulted in a non-significant effect of taxonomic affiliation over sequencing success. As revealed by BLAST against the NCBI non-redundant sequence database, approximately 40% of the sequences obtained corresponded to non-target organisms likely to have been co-extracted with the sponge DNA. Most non-target sequences (∼59%) matched Alpha- (∼39%), Beta- (∼6%) and Gammaproteobacteria (∼14%) strains. The remaining non-target sequences matched annelids (13%), arthropods (∼3%), chordates (∼7%), cnidarians (∼5%), molluscs (2%), gastrotrichs (∼5), sipunculids (∼4%) or uncultured marine organisms, members of the PX clade and florideophyceans (∼3%). E-values for the best matching sequence varied between 0 and 1×10–18 for all blasted contigs (Table S2). Despite the contaminant being co-amplified or preferentially amplified, DNA of sponge origin was present in the extracts as revealed by the amplification of partial 28S rDNA sequences of poriferan origin from the same extracts (results not shown).

**Table 2:** Generalised linear model (binomial errors, logit link) of the effect of sample taxonomic affiliation and sample age over sequencing success.

Variable	Degrees of freedom	Deviance	Residual degrees of freedom	Residual deviance	Probability
	10	18.359	216	296.29	<0.049
Family	12	15.557	204	285.15	N.S.
	16	38.178	250	331.66	0.001
	3	1.209	213	295.08	N.S.
Age category	2	0.700	202	284.45	N.S.
	2	0.024	248	331.64	N.S.
Family	18	21.424	195	273.66	N.S.
*	11	13.882	191	270.57	N.S.
Age category	23	28.424	225	303.21	N.S.

The reported values, are for the core family group (Acarnidae, Ancorinidae, Axinellidae, Dyctionellidae, Dysideidae, Halichondridae, Iotrochotidae, Microcionidae, Mycalidae, Plakinidae, Raspailidae, Tedaniidae, Tetillidae and Thorectidae; upper value) and for this group together with the families Chalinidae, Clionaidae and Suberitidae (middle value), and with the families Chondropsidae, Coelosphaeridae, Crellidae, Desmacellidae, Isodictyidae and Podospongiidae (lower value). N.S. = not significant.

## Discussion

We have presented a first assessment on the progress and technical aspects of the Sponge Barcoding Project. At present, two laboratory workers are capable of processing 576 samples (i.e. 6 96-well plates) a week using the analytical pipeline set for the project. In our experience, subsampling the sponge tissues for extraction is the limiting step in terms of the time needed to process a plate. Subsampling sponge tissue is a time consuming process and it is important that care is taken with this step to ensure that surface contaminants are minimized and that tissues are prepared in small pieces to facilitate the extraction work-flow. After tissue has been subsampled, DNA extraction is completed within hours depending on the worker's experience. This means that DNA extraction, PCR, gel documentation and sequencing for 192 samples (i.e. two 96-well plates) can be done within two working days by one laboratory employee. This modest capacity allows a medium throughput facility to easily barcode large number of samples within short time. Moreover, because the DNA concentration of the extracts is generally high —although this depends greatly on the tissue sample— the barcoding pipeline indirectly results in the establishment of a DNA-bank which can be further used for different purposes.

With respect to PCR success rates the values reported here correspond with published PCR success rates for archival moth specimens when Taq polymerase was used (i.e. <25% PCR success; [19]). We did not observe a general drop in PCR success rates with age, however restricting the analysis to certain families (see Results) revealed a clear relation between PCR success rates and mean sample age. A negative effect of sample age on PCR success rates should not be a surprise if the material used for DNA extraction was not preserved and stored specifically for this purpose, as is the case here. In addition, we have observed consistently low 260/230 absorbance ratios in our extractions. Low 260/230 values have been related to the co-elution of thiocyanate salts [20]; these strong protein denaturants could act synergistically with low-quality DNA to cause PCR failure in these cases. Future sponge barcoding campaigns using fresh tissue or focusing on recent collections preserved specifically for DNA studies could reduce the impact of sample age on PCR success and result in better PCR success values.

In the case of samples yielding DNA of moderate to high quality, a family-specific effect cannot be ruled out as the cause of PCR failure. Our results revealed that PCR success is affected by family membership and that a complex relationship between sample age and taxonomic membership can also influence the performance of the barcoding pipeline (Table 1). The presence of secondary metabolites that could inhibit the PCR reaction is possible in sponges (see [21]), and family or genus specific mismatches in the primer annealing site cannot be discarded. Morphological factors (which are related to taxon membership), such as tissue density or perfusion rates, can influence the rate and quality of specimen fixation and therefore affect the preservation of DNA. We have observed that although tissue subsampling has been standardised, it is particularly difficult to obtain homogeneous DNA concentrations within most DNA extraction plates. High intra-plate variation in DNA concentration hampers the high-throughput downstream processing of the samples, because samples that probably need to be diluted co-exist with low concentration samples that, in all likelihood, will not amplify after dilution. Increasing the volume of the buffer used in the digestion and binding steps of the DNA extraction protocol has helped to reduce intra-plate variability to some extent, but this remains problematic for the high-throughput barcoding of sponges.

Co-amplification or preferential amplification of non-target organisms represents a major obstacle for DNA barcoding (see [22]). We obtained non-target organisms in 40% of the sequenced samples, which, in the context of this study, implied almost a doubling of the relative cost of generating a single sponge barcode. This problem is hard to solve because the complete isolation of contaminating tissues from sponge tissue is usually not possible, and because the phylogenetic origin of the “contaminants” can be diverse. Moreover, cloning is only possible in selected cases as this technique is not compatible with medium- or high-throughput sample processing. Here, we have demonstrated that a sponge DNA extract is actually a complex DNA mixture and can be better thought of as a sponge's holobiont meta-genome. Future work on the design of better primer sets or primer mixtures for sponges based on increased taxonomic sampling should help to improve the efficiency and selectivity of *COI* barcoding for Porifera.

## Materials and Methods

### Tissue samples and DNA extraction

Sponge tissue for this study was subsampled from material deposited at the Queensland Museum (QM; Brisbane, Australia). In brief, small pieces of alcohol preserved sponges were placed in 96-well Eppendorf PCR plates until further processing. The processed material includes representatives from all poriferan classes and demosponge orders, and was collected over the last ∼40 years mainly for classical taxonomy and for inventory purposes; samples were not preserved specifically for DNA analysis.

A number of high-throughput methods for DNA extraction have been published (e.g. [23], [24]). For the Sponge Barcoding Project, we looked for a centrifugation-based method available for 96-well plates. We selected the method proposed by [25] for the Barcoding of Life, which is based on the selective binding of DNA molecules to a fibre-glass membrane (AcroPrep 1 uM glass fiber; Pall 5051) in the presence of high concentrations of Guanidinium Thiocyanate. This method has been shown to give results comparable to commercial alternativese at a fraction of their cost [25]. We optimised the published protocol (see Table 3) to increase the amount of tissue digested and the final DNA concentration (ng/µL). Using this modification, we have extracted a total ∼7400 QM sponge samples to date, covering all poriferan classes and demosponges orders (Table S1). In order to evaluate the amount of DNA obtained, the concentration (ng/µL) of 12 randomly picked extracts per plate was determined using a Nanodrop 1000 spectrophotometer. In total, 14 plates were quantified.

**Table 3:** Modifications to the genomic DNA protocol of Ivanova et al. 2006 used for sponge barcoding.

Protocol Step	Ivanova et al. 2006	This study
**Digestion**	50 uL Lysis mix^1^	200 uL Lysis mix
**Binding**	100 uL Binding mix^2^	400 uL Binding mix
**First washing step**	180 uL Protein wash buffer^3^	200 uL Protein wash buffer
**Second washing step**	750 uL Wash buffer^4^	750 uL Wash buffer
**Elution**	60 uL H_2_O	50–100 uL H_2_O

1 Lysis mix: 100 mM NaCl, 50 mM Tris-HCl pH 8.0, 10 mM EDTA pH 8.0, 0.5% SDS, Proteinase K 10% v/v.

2 Binding buffer: 6 M GuSCN, 20 mM EDTA pH 8.0, 10 mM Tris-HCl pH 6.4, Triton X-100 4% v/v. The Binding mix is a 50% v/v solution of Binding Buffer in ethanol 96%.

3 Protein wash buffer is a 30% v/v solution of Binding Buffer in ethanol 96%.

4 Wash buffer: 50 mM NaCl, 10 mM Tris-HCl pH 7.4, 0.5 mM EDTA pH 8.0, ethanol 60%.

### Amplification and sequencing success of the standard barcoding fragment

The Sponge Barcoding Project focusses initially on sequencing the standard barcoding partition, located at the 5′ end of the mitochondrial cytochrome oxidase subunit 1 [26], [27], to comply with the current convention for metazoan barcoding (but see [28]). We have used COI degenerate primers: dgLCO1490: 5′-GGTCAACAAATCATAAAGAYATYGG-3′; and dgHCO2198: 5′-TAAACTTCAGGGTGACCAAARAAYCA-3′ (Meyer et al. (2005); [29]). Reactions were supplemented with BSA. The Meyer et al. (2005) primer set has been used for sponge barcoding with success [13], [14], [15], thus this primer pair was chosen for the sponge barcoding project pipeline in the absence of more specific alternatives. The amplification program used was a standard three-step PCR with an initial denaturation step of 3 minutes at 94°C followed by 35–40 cycles of 30 seconds at 94°C, 30 seconds at 40°C and 1 minute at 72°C, and a final extension step of 5 minutes at 72°C. Low stringency amplification conditions should result in higher PCR success with the potential disadvantage of poor specificity. However, increasing the annealing temperature of the LCO/HCO primers could result in the undesired, preferential amplification of bacterial over metazoan targets due to a better match of several bacterial strains to the Folmer primers (or derivatives thereof e.g. [22], [29]). Thus, we kept low stringency PCR conditions for the amplification of the standard barcoding fragment.

PCR products were visualised on 1% agarose gels via electrophoresis, and each reaction was categorised as “positive” or “negative”. The relationship between family membership and sample age (years post-collection) on PCR success was evaluated using generalised linear models with binomial errors and logit link. Only families with at least 30 documented PCR reactions were analysed. For the analysis, sample age (years post-collection) was recoded into six age categories (0–5 years, 5–10 years, 10–15 years, 15–20 years, 20–25 years, ≥25 years). Recoding was necessary due to the uneven distribution of sampling effort per family through time. Most families did not have samples in the first and last age categories (0–5 years and ≥25 years), thus these intervals were not used in the analysis. The families Acarnidae, Ancorinidae, Axinellidae, Dyctionellidae, Dysideidae, Halichondridae, Iotrochotidae, Microcionidae, Mycalidae, Plakinidae, Raspailidae, Tedaniidae, Tetillidae and Thorectidae had samples in all analysed age categories (i.e. 5–10 years, 10–15 years, 15–20 years and 20–25 years); PCR success in this core group of families was analysed for all age categories. The families Chalinidae, Clionaidae and Suberitidae did not have any samples collected in the last age category (i.e. 20–25 years) and the families Chondropsidae, Coelosphaeridae, Crellidae, Desmacellidae, Isodictyidae, Podospongiidae lacked samples of age 5–10 years. These two groups of families, together with families in the core group (see above), were independently analysed for the age categories were they had samples available for the analysis.

Sequencing was done after a standard ammonium Acetate–ethanol clean-up [30] using the BigDye 3.1 chemistry (Applied Biosystems) following the protocol provided by the manufacturer and the same primers as used for PCR. Reads were assembled and the contigs were annotated as “contamination” or “sponge” according to the results obtained from BLAST [31] against the non-redundant sequence database of the NCBI. The sequence with the lowest E-value was used to annotate the taxonomic affinity of each query sequence. The effect of taxonomic affiliation and sample age over sequencing success was assessed using Generalised linear models with binomial errors and logit link. For this analysis, the same family groups used for the analysis of PCR success were analysed. Finally, in order to test for the presence of sponge DNA in selected extracts tagged as “contamination”, we amplified ∼1.2 kb of the nuclear 28S rDNA using primers NL4F+NL4R, which tend to preferentially amplify poriferan DNA, and the analytical methods described in [32].

## Supporting Information

Table S1 Number of samples extracted per taxonomic groups.
(DOC)

Table S2 Blast results of sequenced sponge specimens. Match indicate whether the best blast match was a member of the phylum Porifera (Match = 1) or of other phylum (Match = 0). The E-value and the accession number of the best match is provided for each of the examined contigs.
(DOC)
