# Supplementary material for: Barcoding Sponges: An Overview Based on Comprehensive Sampling
Source: PLoS One. 2012 Jul 3;7(7):e39345. doi: 10.1371/journal.pone.0039345 (PMC3389008; doi:10.1371/journal.pone.0039345)
Supplement: Table S1 — Number of samples extracted per taxonomic groups. (DOC) [file pone.0039345.s001.doc]

Table S1: Number of samples extracted per taxonomic groups.

| Taxonomic group | Number of samples extracted |
| --- | --- |
| (Calcarea) | 1 |
| (Lyssacinosida incertae sedis) | 1 |
| Acanthochaetetidae | 15 |
| Acarnidae | 49 |
| Agelasidae | 41 |
| Alectonidae | 12 |
| Ancorinidae | 547 |
| Aphrocallistidae | 3 |
| Aplysinellidae | 95 |
| Aplysinidae | 23 |
| Astroscleridae | 35 |
| Axinellidae | 566 |
| Baeriidae | 1 |
| Callyspongiidae | 177 |
| Calthropellidae | 9 |
| Chalinidae | 93 |
| Chondrillidae | 5 |
| Chondropsidae | 84 |
| Cladorhizidae | 6 |
| Clathrinidae | 10 |
| Clionaidae | 230 |
| Coelosphaeridae | 55 |
| Corallistidae | 30 |
| Crambeidae | 20 |
| Crellidae | 62 |
| Darwinellidae | 107 |
| Dendoricellidae | 2 |
| Desmacellidae | 62 |
| Desmacididae | 15 |
| Desmanthidae | 13 |
| Dictyodendrillidae | 14 |
| Dictyonellidae | 411 |
| Dysideidae | 509 |
| Esperiopsidae | 42 |
| Euretidae | 11 |
| FamilyName | 1 |
| Farreidae | 39 |
| Geodiidae | 99 |
| Grantiidae | 6 |
| Guitarridae | 3 |
| Halichondriidae | 126 |
| Halisarcidae | 12 |
| Hamacanthidae | 1 |
| Hemiasterellidae | 6 |
| Heteropiidae | 2 |
| Heteroxyidae | 116 |
| Hyalonematidae | 19 |
| Hymedesmiidae | 35 |
| Ianthellidae | 315 |
| Iotrochotidae | 83 |
| Irciniidae | 81 |
| Isodictyidae | 58 |
| Isoraphiniidae | 2 |
| Jenkinidae | 1 |
| Latrunculiidae | 17 |
| Lelapiidae | 2 |
| Leucaltidae | 12 |
| Leucettidae | 236 |
| Levinellidae | 23 |
| Macandrewiidae | 1 |
| Metaniidae | 7 |
| Microcionidae | 760 |
| Minchinellidae | 5 |
| Mycalidae | 126 |
| Myxillidae | 7 |
| Niphatidae | 139 |
| Pachastrellidae | 15 |
| Petrosiidae | 328 |
| Pheronematidae | 6 |
| Phloeodictyidae | 85 |
| Phymatellidae | 19 |
| Placospongiidae | 6 |
| Plakinidae | 63 |
| Pleromidae | 12 |
| Podospongiidae | 53 |
| Polymastiidae | 13 |
| Pseudoceratinidae | 35 |
| Raspailiidae | 384 |
| Rhabderemiidae | 8 |
| Rossellidae | 12 |
| Scleritodermidae | 19 |
| Siphoniidae | 1 |
| Soleneiscidae | 22 |
| Spirastrellidae | 13 |
| Spongiidae | 384 |
| Spongillidae | 20 |
| Suberitidae | 71 |
| Sycettidae | 37 |
| Tedaniidae | 77 |
| Tethyidae | 30 |
| Tetillidae | 198 |
| Theonellidae | 4 |
| Thorectidae | 707 |
| Timeidae | 1 |
| Trachycladidae | 16 |
| unassigned | 2 |
| unidentified | 257 |
| Verticillititiidae | 16 |
| Total Result | 8610 |

- the difference with the data reported in the text (~8900 specimens) correspond to taxa for which there was no metadata at the time of analysis. These taxa were treated as missing for the Word-cloud construction.
