# Supplementary material for: Barcoding Sponges: An Overview Based on Comprehensive Sampling
Source: PLoS One. 2012 Jul 3;7(7):e39345. doi: 10.1371/journal.pone.0039345 (PMC3389008; doi:10.1371/journal.pone.0039345)
Supplement: Table S2 — Blast results of sequenced sponge specimens. Match indicate whether the best blast match was a member of the phylum Porifera (Match = 1) or of other phylum (Match = 0). The E-value and the accession number of the best match is provided for each of the examined contigs. (DOC) [file pone.0039345.s002.doc]

Table S2: Blast results of sequenced sponge specimens. Match indicate whether the best blast match was a member of the phylum Porifera (Match = 1) or of other phylum (Match = 0). The E-value and the accession number of the best match is provided for each of the examined contigs.

| Query_Name | Query_length | Phylum | Species | Match | E-val | Matching_accesion |
| --- | --- | --- | --- | --- | --- | --- |
| Contig1 | 684 | Porifera | *Axechina raspailioides* | 1 | 0 | JQ034545 |
| Contig2 | 668 | Porifera | *Hippospongia lachne* | 1 | 0 | EU237484 |
| Contig3 | 652 | Porifera | *Ectyoplasia ferox* | 1 | 0 | HE591462 |
| Contig4 | 699 | Porifera | *Negombata magnifica* | 1 | 0 | AM420314 |
| Contig5 | 708 | Porifera | *Haliclona oculata* | 1 | 0 | HQ379430 |
| Contig6 | 699 | Porifera | *Halichondriid sp.* | 1 | 0 | AY561985 |
| Contig7 | 708 | Porifera | *Iotrochota birotulata* | 1 | 0 | EU237486 |
| Contig8 | 707 | Porifera | *Endectyon fruticosum* | 1 | 0 | JQ034559 |
| Contig9 | 720 | Annelida | *Crucigera sp.* | 0 | 2.00E-168 | HM473344 |
| Contig10 | 654 | Porifera | *Hippospongia lachne* | 1 | 0 | EU237484 |
| Contig11 | 680 | Betaproteobacteria | *Azoarcus sp.* | 0 | 3.00E-147 | AM406670 |
| Contig12 | 685 | Porifera | *Agelas mauritiana* | 1 | 0 | DQ069303 |
| Contig13 | 705 | Porifera | *Agelas axifera* | 1 | 0 | DQ069299 |
| Contig14 | 667 | Porifera | *Agelas sp.* | 1 | 0 | DQ069307 |
| Contig15 | 673 | Porifera | *Agelas schmidti* | 1 | 0 | EU237475 |
| Contig16 | 673 | Porifera | *Agelas clathrodes* | 1 | 0 | DQ075784 |
| Contig17 | 680 | Porifera | *Agelas sp.* | 1 | 0 | DQ069307 |
| Contig18 | 701 | Porifera | *Agelas sventres* | 1 | 0 | DQ075735 |
| Contig19 | 707 | Porifera | *Agelas sventres* | 1 | 0 | DQ075731 |
| Contig20 | 711 | Porifera | *Agelas schmidti* | 1 | 0 | EU237475 |
| Contig21 | 676 | Porifera | *Agelas schmidti* | 1 | 0 | EU237475 |
| Contig22 | 695 | Porifera | *Agelas sventres* | 1 | 0 | DQ075735 |
| Contig23 | 756 | Porifera | *Agelas clathrodes* | 1 | 0 | DQ075784 |
| Contig24 | 710 | Porifera | *Agelas axifera* | 1 | 0 | DQ069299 |
| Contig25 | 699 | Porifera | *Agelas sventres* | 1 | 0 | DQ075735 |
| Contig26 | 699 | Porifera | *Agelas schmidti* | 1 | 0 | EU237475 |
| Contig27 | 648 | Porifera | *Agelas sventres* | 1 | 0 | DQ075731 |
| Contig28 | 690 | Porifera | *Agelas schmidti* | 1 | 0 | EU237475 |
| Contig29 | 708 | Porifera | *Agelas schmidti* | 1 | 0 | EU237475 |
| Contig30 | 645 | Porifera | *Raspaciona aculeata* | 1 | 0 | HQ379415 |
| Contig31 | 702 | Florideophyceae | *Hypnea flexicaulis* | 0 | 0 | FN823052 |
| Contig32 | 663 | Porifera | *Agelas axifera* | 1 | 0 | DQ069299 |
| Contig33 | 708 | Porifera | *Agelas schmidti* | 1 | 0 | EU237475 |
| Contig34 | 591 | Alphaproteobacteria | *Candidatus Pelagibacter ubique HTCC1062* | 0 | 6.00E-155 | CP000084 |
| Contig35 | 707 | Porifera | *Agelas schmidti* | 1 | 0 | EU237475 |
| Contig36 | 647 | Porifera | *Agelas sventres* | 1 | 0 | DQ075731 |
| Contig37 | 708 | Porifera | *Agelas sp.* | 1 | 0 | DQ069307 |
| Contig38 | 746 | Porifera | *Agelas sventres* | 1 | 0 | DQ075735 |
| Contig39 | 709 | Porifera | *Agelas schmidti* | 1 | 0 | EU237475 |
| Contig40 | 694 | Porifera | *Agelas sventres* | 1 | 0 | DQ075731 |
| Contig41 | 701 | Porifera | *Agelas sventres* | 1 | 0 | DQ075735 |
| Contig42 | 197 | Porifera | *Halichondriid sp.* | 1 | 6.00E-066 | AY561985 |
| Contig43 | 710 | Porifera | *Agelas schmidti* | 1 | 0 | EU237475 |
| Contig44 | 708 | Porifera | *Agelas sventres* | 1 | 0 | DQ075735 |
| Contig45 | 699 | Porifera | *Agelas schmidti* | 1 | 0 | EU237475 |
| Contig46 | 690 | Porifera | *Agelas sventres* | 1 | 0 | DQ075735 |
| Contig47 | 623 | Alphaproteobacteria | *Candidatus Pelagibacter ubique HTCC1062* | 0 | 2.00E-110 | CP000084 |
| Contig48 | 681 | Porifera | *Agelas sventres* | 1 | 0 | DQ075731 |
| Contig49 | 709 | Chordata | *Homo sapiens* | 0 | 0 | JQ045057 |
| Contig50 | 694 | Porifera | *Agelas schmidti* | 1 | 0 | EU237475 |
| Contig51 | 708 | Porifera | *Agelas sventres* | 1 | 0 | DQ075735 |
| Contig53 | 715 | Porifera | *Agelas sventres* | 1 | 0 | DQ075735 |
| Contig54 | 708 | Porifera | *Agelas axifera* | 1 | 0 | DQ069299 |
| Contig55 | 700 | Porifera | *Agelas schmidti* | 1 | 0 | EU237475 |
| Contig56 | 689 | Porifera | *Agelas sventres* | 1 | 0 | DQ075735 |
| Contig57 | 665 | Porifera | *Agelas schmidti* | 1 | 0 | EU237475 |
| Contig58 | 644 | Betaproteobacteria | *Chromobacterium violaceum ATCC 12472* | 0 | 5.00E-137 | AE016825 |
| Contig59 | 653 | Porifera | *Clathria schoenus* | 1 | 0 | EF519607 |
| Contig60 | 789 | Porifera | *Protosuberites 'Protosuberites' sp.* | 1 | 9.00E-040 | AY561979 |
| Contig61 | 591 | Alphaproteobacteria | *Mesorhizobium loti* | 0 | 5.00E-073 | BA000012 |
| Contig62 | 700 | Chordata | *Homo sapiens* | 0 | 0 | JQ045043 |
| Contig63 | 691 | Alphaproteobacteria | *Mesorhizobium loti* | 0 | 2.00E-097 | BA000012 |
| Contig64 | 705 | Porifera | *Cymbastela vespertina* | 1 | 0 | JQ034539 |
| Contig65 | 674 | Porifera | *Microciona prolifera* | 1 | 0 | DQ087475 |
| Contig66 | 713 | Porifera | *Microciona prolifera* | 1 | 0 | DQ087475 |
| Contig67 | 586 | Porifera | *Amphimedon compressa* | 1 | 4.00E-176 | EU237474 |
| Contig68 | 671 | Porifera | *Clathria schoenus* | 1 | 0 | EF519607 |
| Contig69 | 688 | Chordata | *Homo sapiens* | 0 | 0 | JF303729 |
| Contig70 | 688 | Porifera | *Halichondriid sp.* | 1 | 0 | AY561985 |
| Contig72 | 687 | Porifera | *Monanchora arbuscula* | 1 | 0 | EF519645 |
| Contig73 | 676 | Sipuncula | *Phascolosoma esculenta* | 0 | 1.00E-150 | EF521190 |
| Contig74 | 698 | Chordata | *Homo sapiens* | 0 | 0 | JQ045043 |
| Contig75 | 684 | Porifera | *Endectyon delaubenfelsi* | 1 | 0 | HQ379412 |
| Contig76 | 669 | Porifera | *Tethya actinia* | 1 | 0 | AY320033 |
| Contig77 | 696 | Porifera | *Microciona prolifera* | 1 | 0 | DQ087475 |
| Contig78 | 683 | Porifera | *Neofibularia hartmani* | 1 | 0 | JF773145 |
| Contig79 | 677 | Sipuncula | *Phascolosoma esculenta* | 0 | 6.00E-130 | EF521190 |
| Contig80 | 699 | Porifera | *Iotrochota birotulata* | 1 | 0 | EU237486 |
| Contig81 | 688 | Porifera | *Hippospongia lachne* | 1 | 0 | EU237484 |
| Contig82 | 670 | Porifera | *Tethya actinia* | 1 | 0 | AY320033 |
| Contig83 | 653 | Annelida | *Crucigera sp.* | 0 | 2.00E-143 | HM473344 |
| Contig84 | 656 | Porifera | *Triptolemma intextum* | 1 | 0 | HM592710 |
| Contig85 | 660 | Gammaproteobacteria | *Pseudomonas fluorescens Pf0-1* | 0 | 6.00E-035 | CP000094 |
| Contig86 | 694 | Gammaproteobacteria | *Marinobacter hydrocarbonoclasticus ATCC 49840* | 0 | 3.00E-071 | FO203363 |
| Contig87 | 649 | Gammaproteobacteria | *Methylococcus capsulatus str. Bath* | 0 | 7.00E-066 | AE017282 |
| Contig88 | 679 | Gastrotricha | *Tetranchyroderma sp.3* | 0 | 1.00E-082 | JF432035 |
| Contig89 | 665 | Porifera | *Triptolemma intextum* | 1 | 9.00E-128 | HM592710 |
| Contig90 | 680 | Porifera | *Pachymatisma johnstonia* | 1 | 0 | EF564341 |
| Contig91 | 730 | Gammaproteobacteria | *Marinobacter adhaerens* | 0 | 2.00E-080 | CP001978 |
| Contig92 | 701 | Gastrotricha | *Tetranchyroderma sp.3* | 0 | 1.00E-082 | JF432035 |
| Contig93 | 698 | Gammaproteobacteria | *Marinobacter hydrocarbonoclasticus ATCC 49840* | 0 | 2.00E-086 | FO203363 |
| Contig94 | 772 | Alphaproteobacteria | *Sinorhizobium meliloti* | 0 | 0 | CP002740 |
| Contig95 | 698 | Porifera | *Ecionemia megastylifera* | 1 | 0 | AY561980 |
| Contig96 | 697 | Porifera | *Pachymatisma johnstonia* | 1 | 0 | EF564341 |
| Contig97 | 709 | Porifera | *Geodia vaubani* | 1 | 0 | EU442202 |
| Contig98 | 639 | Betaproteobacteria | *Chromobacterium violaceum ATCC 12472* | 0 | 3.00E-127 | AE016825 |
| Contig99 | 689 | Alphaproteobacteria | *Polymorphum gilvum SL003B-26A1* | 0 | 9.00E-128 | CP002568 |
| Contig100 | 724 | Gastrotricha | *Tetranchyroderma sp.3* | 0 | 9.00E-084 | JF432035 |
| Contig101 | 706 | Porifera | *Pachymatisma johnstonia* | 1 | 0 | EF564341 |
| Contig102 | 699 | Porifera | *Pachymatisma johnstonia* | 1 | 0 | EF564341 |
| Contig103 | 656 | Alphaproteobacteria | *Polymorphum gilvum SL003B-26A1* | 0 | 9.00E-115 | CP002568 |
| Contig104 | 703 | Gammaproteobacteria | *Marinobacter adhaerens* | 0 | 1.00E-081 | CP001978 |
| Contig105 | 702 | Gammaproteobacteria | *Marinobacter hydrocarbonoclasticus ATCC 49840* | 0 | 1.00E-089 | FO203363 |
| Contig106 | 708 | Porifera | *Pachymatisma johnstonia* | 1 | 0 | EF564341 |
| Contig107 | 681 | Porifera | *Pachymatisma johnstonia* | 1 | 0 | EF564341 |
| Contig108 | 706 | Gammaproteobacteria | *Marinobacter hydrocarbonoclasticus ATCC 49840* | 0 | 5.00E-074 | FO203363 |
| Contig109 | 704 | Gastrotricha | *Tetranchyroderma sp.3* | 0 | 7.00E-085 | JF432035 |
| Contig110 | 713 | Gammaproteobacteria | *Marinobacter hydrocarbonoclasticus ATCC 49840* | 0 | 4.00E-088 | FO203363 |
| Contig111 | 701 | Gastrotricha | *Tetranchyroderma sp.3* | 0 | 7.00E-085 | JF432035 |
| Contig112 | 718 | Porifera | *Geodia vaubani* | 1 | 0 | EU442202 |
| Contig113 | 713 | Porifera | *Pachymatisma johnstonia* | 1 | 0 | EF564341 |
| Contig114 | 707 | Porifera | *Pachymatisma johnstonia* | 1 | 0 | EF564341 |
| Contig115 | 559 | Betaproteobacteria | *Azoarcus sp.* | 0 | 2.00E-096 | AM406670 |
| Contig116 | 722 | Alphaproteobacteria | *Polymorphum gilvum SL003B-26A1* | 0 | 5.00E-157 | CP002568 |
| Contig118 | 843 | Gastrotricha | *Tetranchyroderma sp.3* | 0 | 2.00E-087 | JF432035 |
| Contig119 | 715 | Alphaproteobacteria | *Pseudovibrio sp.* | 0 | 1.00E-139 | CP003147 |
| Contig120 | 701 | Porifera | *Pachymatisma johnstonia* | 1 | 0 | EF564341 |
| Contig121 | 707 | Porifera | *Pachymatisma johnstonia* | 1 | 0 | EF564341 |
| Contig122 | 697 | Porifera | *Pachymatisma johnstonia* | 1 | 0 | EF564341 |
| Contig123 | 706 | Porifera | *Ecionemia megastylifera* | 1 | 0 | AY561980 |
| Contig124 | 726 | Alphaproteobacteria | *Polymorphum gilvum SL003B-26A1* | 0 | 5.00E-157 | CP002568 |
| Contig125 | 695 | Gastrotricha | *Tetranchyroderma sp.3* | 0 | 3.00E-084 | JF432035 |
| Contig126 | 682 | Alphaproteobacteria | *Polymorphum gilvum SL003B-26A1* | 0 | 3.00E-114 | CP002568 |
| Contig127 | 697 | Gammaproteobacteria | *Marinobacter hydrocarbonoclasticus ATCC 49840* | 0 | 3.00E-084 | FO203363 |
| Contig128 | 704 | Gastrotricha | *Tetranchyroderma sp.3* | 0 | 2.00E-040 | JF432035 |
| Contig129 | 707 | Porifera | *Pachymatisma johnstonia* | 1 | 0 | EF564341 |
| Contig130 | 707 | Porifera | *Pachymatisma johnstonia* | 1 | 0 | EF564341 |
| Contig131 | 706 | Porifera | *Pachymatisma johnstonia* | 1 | 0 | EF564341 |
| Contig132 | 620 | Betaproteobacteria | *Azoarcus sp.* | 0 | 3.00E-095 | AM406670 |
| Contig133 | 693 | Porifera | *Pachymatisma normani* | 1 | 9.00E-179 | EF564329 |
| Contig134 | 723 | Alphaproteobacteria | *Polymorphum gilvum SL003B-26A1* | 0 | 2.00E-154 | CP002568 |
| Contig135 | 722 | Alphaproteobacteria | *Polymorphum gilvum SL003B-26A1* | 0 | 5.00E-157 | CP002568 |
| Contig136 | 696 | Gammaproteobacteria | *Marinobacter adhaerens* | 0 | 7.00E-079 | CP001978 |
| Contig137 | 704 | Porifera | *Pachymatisma johnstonia* | 1 | 0 | EF564341 |
| Contig138 | 695 | Porifera | *Pachymatisma johnstonia* | 1 | 0 | EF564341 |
| Contig139 | 666 | Betaproteobacteria | *Azoarcus sp.* | 0 | 5.00E-144 | AM406670 |
| Contig140 | 664 | Porifera | *Clathria schoenus* | 1 | 0 | EF519607 |
| Contig141 | 695 | Alphaproteobacteria | *Bradyrhizobium japonicum USDA 110* | 0 | 2.00E-092 | BA000040 |
| Contig142 | 671 | Porifera | *Microciona prolifera* | 1 | 0 | AJ704978 |
| Contig143 | 669 | Porifera | *Clathria schoenus* | 1 | 0 | EF519607 |
| Contig144 | 195 | Porifera | *Halichondriid sp.* | 1 | 7.00E-072 | AY561985 |
| Contig145 | 1056 | Alphaproteobacteria | *Candidatus Pelagibacter ubique HTCC1062* | 0 | 4.00E-027 | CP000084 |
| Contig146 | 536 | Porifera | *Thrinacophora cervicornis* | 1 | 0 | JQ034586 |
| Contig147 | 196 | Porifera | *Halichondriid sp.* | 1 | 7.00E-072 | AY561985 |
| Contig148 | 679 | Porifera | *Negombata magnifica* | 1 | 0 | AM420314 |
| Contig149 | 720 | Alphaproteobacteria | *Dinoroseobacter shibae DFL 12* | 0 | 3.00E-153 | CP000830 |
| Contig150 | 708 | Porifera | *Monanchora arbuscula* | 1 | 0 | EF519645 |
| Contig151 | 698 | Porifera | *Echinodictyum clathrioides* | 1 | 0 | JQ034556 |
| Contig152 | 716 | Alphaproteobacteria | *Dinoroseobacter shibae DFL 12* | 0 | 3.00E-146 | CP000830 |
| Contig153 | 681 | Porifera | *Thrinacophora cervicornis* | 1 | 0 | JQ034586 |
| Contig154 | 196 | Porifera | *Halichondriid sp.* | 1 | 2.00E-072 | AY561985 |
| Contig155 | 722 | Porifera | *Echinodictyum mesenterinum* | 1 | 6.00E-067 | JQ034558 |
| Contig156 | 711 | Alphaproteobacteria | *Dinoroseobacter shibae DFL 12* | 0 | 2.00E-150 | CP000830 |
| Contig157 | 670 | Porifera | *Clathria schoenus* | 1 | 0 | EF519607 |
| Contig158 | 700 | Annelida | *Crucigera sp.* | 0 | 2.00E-169 | HM473344 |
| Contig159 | 726 | Alphaproteobacteria | *Rhodobacter sphaeroides ATCC 17025* | 0 | 4.00E-152 | CP000661 |
| Contig160 | 675 | Alphaproteobacteria | *Dinoroseobacter shibae DFL 12* | 0 | 2.00E-141 | CP000830 |
| Contig161 | 675 | Porifera | *Monanchora arbuscula* | 1 | 0 | EF519645 |
| Contig162 | 772 | Alphaproteobacteria | *Dinoroseobacter shibae DFL 12* | 0 | 2.00E-136 | CP000830 |
| Contig163 | 692 | Porifera | *Microciona prolifera* | 1 | 0 | DQ087475 |
| Contig164 | 645 | Alphaproteobacteria | *Polymorphum gilvum SL003B-26A1* | 0 | 5.00E-099 | CP002568 |
| Contig165 | 941 | Alphaproteobacteria | *Bradyrhizobium sp.* | 0 | 4.00E-026 | AP012279 |
| Contig166 | 664 | Porifera | *Microciona prolifera* | 1 | 0 | AJ704978 |
| Contig167 | 589 | Porifera | *Microciona prolifera* | 1 | 5.00E-175 | DQ087475 |
| Contig168 | 710 | Porifera | *Negombata magnifica* | 1 | 0 | AM420314 |
| Contig169 | 704 | Annelida | *Crucigera sp.* | 0 | 8.00E-148 | HM473344 |
| Contig170 | 700 | Porifera | *Hemiasterella sp.* | 1 | 0 | AY561977 |
| Contig171 | 700 | Sipuncula | *Phascolosoma esculenta* | 0 | 3.00E-121 | EF521190 |
| Contig172 | 658 | Alphaproteobacteria | *Polymorphum gilvum SL003B-26A1* | 0 | 2.00E-073 | CP002568 |
| Contig173 | 686 | Porifera | *Rhabdastrella globostellata* | 1 | 0 | HM592683 |
| Contig174 | 628 | Porifera | *Rhabdastrella globostellata* | 1 | 0 | HM592683 |
| Contig175 | 679 | Porifera | *Hemiasterella sp.* | 1 | 0 | AY561977 |
| Contig176 | 708 | Arthropoda | *Brachyura sp.* | 0 | 0 | HM464349 |
| Contig177 | 708 | Porifera | *Hemiasterella sp.* | 1 | 0 | AY561977 |
| Contig178 | 705 | Porifera | *Agelas schmidti* | 1 | 0 | EU237475 |
| Contig179 | 709 | Porifera | *Hemiasterella sp.* | 1 | 0 | AY561977 |
| Contig180 | 707 | Porifera | *Hemiasterella sp.* | 1 | 0 | AY561977 |
| Contig181 | 638 | Porifera | *Ecionemia megastylifera* | 1 | 0 | AY561980 |
| Contig183 | 697 | Porifera | *Agelas sventres* | 1 | 0 | DQ075735 |
| Contig184 | 607 | Porifera | *Rhabdastrella globostellata* | 1 | 0 | HM592683 |
| Contig185 | 707 | Porifera | *Rhabdastrella globostellata* | 1 | 0 | HM592683 |
| Contig187 | 708 | Porifera | *Hemiasterella sp.* | 1 | 0 | AY561977 |
| Contig188 | 700 | Porifera | *Rhabdastrella globostellata* | 1 | 0 | HM592683 |
| Contig189 | 706 | Porifera | *Agelas sventres* | 1 | 0 | DQ075735 |
| Contig190 | 709 | Alphaproteobacteria | *Mesorhizobium opportunistum* | 0 | 1.00E-089 | CP002279 |
| Contig191 | 706 | Porifera | *Agelas cf. sventres INV-POR 1011* | 1 | 0 | DQ075755 |
| Contig192 | 708 | Porifera | *Agelas sventres* | 1 | 0 | DQ075731 |
| Contig193 | 614 | Porifera | *Rhabdastrella globostellata* | 1 | 0 | HM592683 |
| Contig194 | 714 | Sipuncula | *Phascolosoma esculenta* | 0 | 1.00E-125 | EF521190 |
| Contig195 | 706 | Porifera | *Ecionemia megastylifera* | 1 | 0 | AY561980 |
| Contig196 | 644 | Porifera | *Ecionemia megastylifera* | 1 | 0 | AY561980 |
| Contig197 | 597 | Sipuncula | *Phascolosoma esculenta* | 0 | 3.00E-083 | EF521190 |
| Contig198 | 708 | Porifera | *Agelas sp.* | 1 | 0 | DQ069307 |
| Contig199 | 604 | Porifera | *Agelas sventres* | 1 | 0 | DQ075731 |
| Contig200 | 706 | Porifera | *Rhabdastrella globostellata* | 1 | 0 | HM592683 |
| Contig201 | 614 | Porifera | *Hemiasterella sp.* | 1 | 0 | AY561977 |
| Contig202 | 667 | Betaproteobacteria | *Azoarcus sp.* | 0 | 5.00E-144 | AM406670 |
| Contig203 | 652 | Alphaproteobacteria | *Nitrobacter hamburgensis* | 0 | 2.00E-065 | CP000319 |
| Contig204 | 695 | Porifera | *Agelas sventres* | 1 | 0 | DQ075735 |
| Contig205 | 695 | Porifera | *Agelas sventres* | 1 | 0 | DQ075735 |
| Contig206 | 697 | Porifera | *Agelas axifera* | 1 | 0 | DQ069299 |
| Contig207 | 701 | Porifera | *Agelas sp.* | 1 | 0 | DQ069307 |
| Contig208 | 697 | Porifera | *Agelas schmidti* | 1 | 0 | EU237475 |
| Contig209 | 686 | Alphaproteobacteria | *Candidatus Pelagibacter sp.* | 0 | 7.00E-047 | CP002511 |
| Contig210 | 708 | Porifera | *Agelas sventres* | 1 | 0 | DQ075731 |
| Contig211 | 658 | Porifera | *Agelas schmidti* | 1 | 0 | EU237475 |
| Contig212 | 697 | Porifera | *Agelas sventres* | 1 | 0 | DQ075735 |
| Contig213 | 689 | Porifera | *Agelas sventres* | 1 | 0 | DQ075735 |
| Contig214 | 707 | Porifera | *Agelas schmidti* | 1 | 0 | EU237475 |
| Contig215 | 695 | Porifera | *Agelas schmidti* | 1 | 0 | EU237475 |
| Contig216 | 687 | Porifera | *Agelas sventres* | 1 | 0 | DQ075731 |
| Contig217 | 711 | Porifera | *Agelas sp.* | 1 | 0 | DQ069307 |
| Contig218 | 707 | Porifera | *Agelas axifera* | 1 | 0 | DQ069299 |
| Contig219 | 693 | Alphaproteobacteria | *Pseudovibrio sp.* | 0 | 1.00E-081 | CP003147 |
| Contig221 | 710 | Porifera | *Agelas nakamurai* | 1 | 0 | DQ069305 |
| Contig222 | 713 | Porifera | *Agelas schmidti* | 1 | 0 | EU237475 |
| Contig223 | 707 | Porifera | *Agelas axifera* | 1 | 0 | DQ069299 |
| Contig224 | 697 | Porifera | *Agelas schmidti* | 1 | 0 | EU237475 |
| Contig225 | 757 | Alphaproteobacteria | *Dinoroseobacter shibae DFL 12* | 0 | 2.00E-142 | CP000830 |
| Contig226 | 695 | Porifera | *Agelas nakamurai* | 1 | 0 | DQ069305 |
| Contig227 | 714 | Porifera | *Agelas sp.* | 1 | 0 | DQ069307 |
| Contig228 | 755 | Porifera | *Agelas sventres* | 1 | 0 | DQ075735 |
| Contig229 | 712 | Porifera | *Agelas sp.* | 1 | 0 | DQ069307 |
| Contig230 | 709 | Porifera | *Agelas axifera* | 1 | 0 | DQ069299 |
| Contig231 | 685 | Betaproteobacteria | *Azoarcus sp.* | 0 | 9.00E-147 | AM406670 |
| Contig232 | 707 | Porifera | *Agelas nakamurai* | 1 | 0 | DQ069305 |
| Contig233 | 709 | Porifera | *Agelas clathrodes* | 1 | 0 | DQ075702 |
| Contig234 | 765 | Porifera | *Agelas sventres* | 1 | 0 | DQ075731 |
| Contig235 | 708 | Porifera | *Agelas schmidti* | 1 | 0 | EU237475 |
| Contig236 | 826 | Porifera | *Agelas sventres* | 1 | 0 | DQ075735 |
| Contig238 | 666 | Porifera | *Negombata magnifica* | 1 | 0 | AM420314 |
| Contig239 | 693 | Porifera | *Monanchora arbuscula* | 1 | 0 | EF519645 |
| Contig241 | 722 | Alphaproteobacteria | *Ruegeria pomeroyi DSS-3* | 0 | 6.00E-156 | CP000031 |
| Contig242 | 676 | Alphaproteobacteria | *Rhodobacter sphaeroides ATCC 17025* | 0 | 2.00E-155 | CP000661 |
| Contig243 | 673 | Porifera | *Microciona prolifera* | 1 | 0 | DQ087475 |
| Contig244 | 706 | Porifera | *Clathria schoenus* | 1 | 0 | EF519607 |
| Contig245 | 710 | Porifera | *Iotrochota birotulata* | 1 | 0 | EU237486 |
| Contig246 | 709 | Porifera | *Monanchora arbuscula* | 1 | 0 | EF519645 |
| Contig247 | 708 | Porifera | *Iotrochota birotulata* | 1 | 0 | EU237486 |
| Contig248 | 704 | Porifera | *Negombata magnifica* | 1 | 0 | AM420314 |
| Contig249 | 508 | Arthropoda | *Onymocoris izzardi* | 0 | 1.00E-079 | AY253122 |
| Contig250 | 699 | Porifera | *Iotrochota birotulata* | 1 | 0 | EU237486 |
| Contig251 | 742 | Alphaproteobacteria | *Ruegeria pomeroyi DSS-3* | 0 | 1.00E-146 | CP000031 |
| Contig252 | 697 | Chordata | *Lutjanus johnii* | 0 | 0 | EU502682 |
| Contig253 | 698 | Annelida | *Crucigera sp.* | 0 | 5.00E-150 | HM473344 |
| Contig255 | 710 | Porifera | *Negombata magnifica* | 1 | 0 | AM420314 |
| Contig256 | 750 | Bryozoa | *Vesiculariidae gen. n. sp.* | 0 | 0 | JN680992 |
| Contig257 | 696 | Porifera | *Iotrochota birotulata* | 1 | 0 | EU237486 |
| Contig258 | 695 | Porifera | *Iotrochota birotulata* | 1 | 0 | EU237486 |
| Contig259 | 709 | Porifera | *Microciona prolifera* | 1 | 0 | DQ087475 |
| Contig260 | 698 | Annelida | *Crucigera sp.* | 0 | 5.00E-150 | HM473344 |
| Contig261 | 760 | uncultured marine organism | *environmental samples uncultured marine organism* | 0 | 0 | GQ234900 |
| Contig262 | 738 | Alphaproteobacteria | *Rhodobacter sphaeroides ATCC 17029* | 0 | 8.00E-161 | CP000577 |
| Contig263 | 696 | Cnidaria | *Savalia savaglia* | 0 | 0 | DQ825686 |
| Contig264 | 707 | Porifera | *Diacarnus spinipoculum* | 1 | 0 | AY561975 |
| Contig265 | 700 | Annelida | *Crucigera sp.* | 0 | 0 | HM473344 |
| Contig266 | 707 | Cnidaria | *Savalia savaglia* | 0 | 0 | DQ825686 |
| Contig267 | 711 | Porifera | *Negombata magnifica* | 1 | 0 | AM420314 |
| Contig268 | 695 | Porifera | *Negombata magnifica* | 1 | 0 | AM420314 |
| Contig269 | 711 | Porifera | *Iotrochota birotulata* | 1 | 0 | EU237486 |
| Contig270 | 712 | Porifera | *Clathria schoenus* | 1 | 0 | EF519607 |
| Contig271 | 683 | Porifera | *Plocamionida sp.* | 1 | 0 | FR687230 |
| Contig272 | 751 | Porifera | *Microciona prolifera* | 1 | 0 | AJ704978 |
| Contig273 | 697 | Betaproteobacteria | *Azoarcus sp.* | 0 | 9.00E-147 | AM406670 |
| Contig274 | 620 | Porifera | *Ianthella basta* | 1 | 0 | JF915542 |
| Contig275 | 708 | Porifera | *Ianthella basta* | 1 | 0 | JF915542 |
| Contig276 | 707 | Porifera | *Ianthella basta* | 1 | 0 | JF915542 |
| Contig277 | 694 | Porifera | *Ianthella basta* | 1 | 0 | JF915539 |
| Contig278 | 680 | Porifera | *Ianthella basta* | 1 | 0 | JF915542 |
| Contig279 | 681 | Porifera | *Ianthella basta* | 1 | 0 | JF915539 |
| Contig280 | 718 | Porifera | *Ianthella basta* | 1 | 0 | JF915542 |
| Contig281 | 691 | Porifera | *Ianthella basta* | 1 | 0 | JF915539 |
| Contig282 | 706 | Porifera | *Ianthella basta* | 1 | 0 | JF915542 |
| Contig283 | 603 | Porifera | *Ianthella basta* | 1 | 0 | JF915543 |
| Contig284 | 697 | Porifera | *Ianthella basta* | 1 | 0 | JF915539 |
| Contig285 | 523 | Alphaproteobacteria | *Dinoroseobacter shibae DFL 12* | 0 | 3.00E-100 | CP000830 |
| Contig286 | 681 | Porifera | *Ianthella basta* | 1 | 0 | JF915539 |
| Contig287 | 696 | Porifera | *Ianthella basta* | 1 | 0 | JF915539 |
| Contig288 | 694 | Porifera | *Ianthella basta* | 1 | 0 | JF915539 |
| Contig289 | 588 | Porifera | *Ianthella basta* | 1 | 0 | JF915539 |
| Contig290 | 692 | Porifera | *Ianthella basta* | 1 | 0 | JF915539 |
| Contig291 | 699 | Porifera | *Ianthella basta* | 1 | 0 | JF915539 |
| Contig292 | 700 | Porifera | *Ianthella basta* | 1 | 0 | JF915539 |
| Contig293 | 705 | Porifera | *Ianthella basta* | 1 | 0 | JF915542 |
| Contig294 | 660 | Alphaproteobacteria | *Azospirillum brasilense* | 0 | 2.00E-124 | HE577327 |
| Contig295 | 518 | Porifera | *Ianthella basta* | 1 | 0 | JF915543 |
| Contig296 | 620 | Porifera | *Ianthella basta* | 1 | 0 | JF915543 |
| Contig297 | 699 | Porifera | *Ianthella basta* | 1 | 0 | JF915542 |
| Contig298 | 691 | Porifera | *Ianthella basta* | 1 | 0 | JF915543 |
| Contig299 | 685 | Porifera | *Ianthella basta* | 1 | 0 | JF915539 |
| Contig300 | 635 | Porifera | *Ianthella basta* | 1 | 0 | JF915543 |
| Contig301 | 692 | Porifera | *Ianthella basta* | 1 | 0 | JF915539 |
| Contig302 | 694 | Porifera | *Ianthella basta* | 1 | 0 | JF915539 |
| Contig303 | 624 | Porifera | *Ianthella basta* | 1 | 0 | JF915539 |
| Contig304 | 670 | Porifera | *Ianthella basta* | 1 | 0 | JF915539 |
| Contig305 | 705 | Porifera | *Ianthella basta* | 1 | 0 | JF915539 |
| Contig306 | 705 | Porifera | *Ianthella basta* | 1 | 0 | JF915542 |
| Contig307 | 638 | Porifera | *Ianthella basta* | 1 | 0 | JF915539 |
| Contig308 | 700 | Porifera | *Ianthella basta* | 1 | 0 | JF915539 |
| Contig309 | 503 | Porifera | *Ianthella basta* | 1 | 0 | JF915543 |
| Contig310 | 706 | Porifera | *Ianthella basta* | 1 | 0 | JF915539 |
| Contig311 | 695 | Porifera | *Ianthella basta* | 1 | 0 | JF915539 |
| Contig312 | 695 | Porifera | *Ianthella basta* | 1 | 0 | JF915539 |
| Contig314 | 645 | Chordata | *Homo sapiens* | 0 | 0 | JQ245806 |
| Contig315 | 644 | Porifera | *Topsentia ophiraphidites* | 1 | 0 | EU237482 |
| Contig316 | 619 | Porifera | *Microciona prolifera* | 1 | 0 | DQ087475 |
| Contig317 | 683 | Alphaproteobacteria | *Ruegeria pomeroyi DSS-3* | 0 | 0 | CP000031 |
| Contig318 | 684 | Porifera | *Topsentia ophiraphidites* | 1 | 0 | EU237482 |
| Contig319 | 688 | Porifera | *Neofibularia hartmani* | 1 | 0 | JF773145 |
| Contig320 | 689 | Porifera | *Topsentia ophiraphidites* | 1 | 0 | EU237482 |
| Contig321 | 706 | Porifera | *Ceratoporella nicholsoni* | 1 | 0 | DQ075775 |
| Contig322 | 710 | Porifera | *Clathria schoenus* | 1 | 0 | EF519607 |
| Contig323 | 708 | Porifera | *Neamphius huxleyi* | 1 | 0 | HM592682 |
| Contig324 | 697 | Porifera | *Clathria schoenus* | 1 | 0 | EF519607 |
| Contig325 | 492 | Porifera | *Halichondriid sp.* | 1 | 2.00E-046 | AY561985 |
| Contig326 | 694 | Porifera | *Halichondriid sp.* | 1 | 0 | AY561985 |
| Contig327 | 684 | Gammaproteobacteria | *Glaciecola sp.* | 0 | 1.00E-050 | CP002526 |
| Contig328 | 697 | Porifera | *Amphimedon compressa* | 1 | 0 | EU237474 |
| Contig329 | 686 | Porifera | *Clathria oxeota* | 1 | 0 | EF519605 |
| Contig330 | 611 | Gammaproteobacteria | *Candidatus Ruthia magnifica str. Cm (Calyptogena magnifica)* | 0 | 6.00E-041 | CP000488 |
| Contig331 | 708 | Porifera | *Astrosclera willeyana* | 1 | 0 | AY561969 |
| Contig332 | 684 | Porifera | *Halichondriid sp.* | 1 | 0 | AY561985 |
| Contig333 | 638 | Porifera | *Echinodictyum cancellatum* | 1 | 0 | JQ034555 |
| Contig334 | 696 | Porifera | *Plocamionida sp.* | 1 | 0 | FR687230 |
| Contig335 | 681 | Porifera | *Cliona celata* | 1 | 2.00E-048 | HM999035 |
| Contig336 | 707 | Porifera | *Astrosclera willeyana* | 1 | 0 | AY561969 |
| Contig337 | 710 | Porifera | *Pachymatisma johnstonia* | 1 | 0 | EF564341 |
| Contig338 | 696 | Porifera | *Halichondriid sp.* | 1 | 0 | AY561985 |
| Contig339 | 710 | Porifera | *Axinella corrugata* | 1 | 0 | AY791693 |
| Contig340 | 700 | Gammaproteobacteria | *Alkalilimnicola ehrlichii MLHE-1* | 0 | 3.00E-153 | CP000453 |
| Contig341 | 650 | Gammaproteobacteria | *Alkalilimnicola ehrlichii MLHE-1* | 0 | 2.00E-117 | CP000453 |
| Contig342 | 725 | Alphaproteobacteria | *Azospirillum brasilense* | 0 | 5.00E-131 | HE577327 |
| Contig343 | 716 | Alphaproteobacteria | *Rhodobacter sphaeroides ATCC 17029* | 0 | 3.00E-160 | CP000577 |
| Contig344 | 677 | Porifera | *Tethyid sp.* | 1 | 0 | AY561986 |
| Contig345 | 643 | Gammaproteobacteria | *Glaciecola sp.* | 0 | 3.00E-082 | CP002526 |
| Contig346 | 719 | Porifera | *Axinella infundibuliformis* | 1 | 0 | HQ379410 |
| Contig354 | 707 | Porifera | *Agelas sventres* | 1 | 0 | DQ075735 |
| Contig355 | 716 | Porifera | *Thenea levis* | 1 | 0 | HM592747 |
| Contig356 | 693 | Porifera | *Rhabdastrella globostellata* | 1 | 0 | HM592683 |
| Contig486 | 707 | Porifera | *Geodia papyracea* | 1 | 0 | AY561961 |
| Contig487 | 699 | Porifera | *Thenea levis* | 1 | 0 | HM592747 |
| Contig488 | 710 | Porifera | *Pachymatisma johnstonia* | 1 | 0 | EF564341 |
| Contig489 | 687 | Porifera | *Stelletta sp.* | 1 | 0 | FJ711644 |
| Contig490 | 694 | Porifera | *Neamphius huxleyi* | 1 | 0 | HM592682 |
| Contig491 | 687 | Porifera | *Pachymatisma johnstonia* | 1 | 0 | EF564341 |
| Contig492 | 682 | Porifera | *Ecionemia sp.* | 1 | 0 | HM592725 |
| Contig493 | 677 | Porifera | *Ecionemia sp.* | 1 | 0 | HM592725 |
| Contig494 | 708 | Porifera | *Agelas schmidti* | 1 | 0 | EU237475 |
| Contig495 | 688 | Porifera | *Eunapius subterraneus* | 1 | 0 | GU086203 |
| Contig496 | 708 | Porifera | *Rhabdastrella intermedia* | 1 | 0 | HM592726 |
| Contig497 | 686 | Porifera | *Agelas axifera* | 1 | 0 | DQ069299 |
| Contig498 | 695 | Porifera | *Haliclona amphioxa* | 1 | 0 | AJ843892 |
| Contig499 | 710 | Porifera | *Rhabdastrella cordata* | 1 | 0 | HM592727 |
| Contig500 | 705 | Porifera | *Ecionemia megastylifera* | 1 | 0 | AY561980 |
| Contig501 | 603 | Porifera | *Ecionemia sp.* | 1 | 0 | HM592725 |
| Contig502 | 708 | Porifera | *Ancorina sp.* | 1 | 0 | HM592744 |
| Contig503 | 652 | Porifera | *Agelas axifera* | 1 | 0 | DQ069299 |
| Contig347 | 709 | Porifera | *Ianthella basta* | 1 | 0 | JF915539 |
| Contig348 | 675 | Gammaproteobacteria | *Alkalilimnicola ehrlichii MLHE-1* | 0 | 3.00E-152 | CP000453 |
| Contig349 | 691 | Annelida | *Chloeia parva* | 0 | 1.00E-126 | EU352320 |
| Contig350 | 713 | Porifera | *Cymbastela vespertina* | 1 | 0 | JQ034539 |
| Contig351 | 698 | Porifera | *Cymbastela vespertina* | 1 | 0 | JQ034539 |
| Contig352 | 709 | Mollusca | *Patelloida striata* | 0 | 1.00E-114 | AB161589 |
| Contig353 | 547 | Chordata | *Gadus chalcogrammus* | 0 | 1.00E-018 | EU266376 |
| Contig357 | 860 | Porifera | *Xestospongia muta* | 1 | 0 | EU237490 |
| Contig358 | 684 | Gammaproteobacteria | *Methylococcus capsulatus str. Bath* | 0 | 2.00E-130 | AE017282 |
| Contig359 | 699 | Annelida | *Haplosyllis spongicola* | 0 | 2.00E-118 | EF123751 |
| Contig360 | 696 | Porifera | *Axinella infundibuliformis* | 1 | 0 | HQ379410 |
| Contig361 | 708 | Porifera | *Callyspongia sp.* | 1 | 0 | JN242195 |
| Contig362 | 699 | Porifera | *Echinodictyum cancellatum* | 1 | 0 | JQ034555 |
| Contig363 | 707 | Porifera | *Protosuberites 'Protosuberites' sp.* | 1 | 0 | AY561979 |
| Contig364 | 741 | Gammaproteobacteria | *Alkalilimnicola ehrlichii MLHE-1* | 0 | 2.00E-142 | CP000453 |
| Contig365 | 703 | Porifera | *Neopetrosia seriata* | 1 | 0 | JN242213 |
| Contig366 | 707 | Chordata | *Homo sapiens* | 0 | 0 | JQ045043 |
| Contig367 | 690 | Alphaproteobacteria | *Candidatus Puniceispirillum marinum IMCC1322* | 0 | 8.00E-148 | CP001751 |
| Contig368 | 708 | Porifera | *Phakellia carduus* | 1 | 0 | JQ034526 |
| Contig369 | 698 | Gammaproteobacteria | *Alkalilimnicola ehrlichii MLHE-1* | 0 | 1.00E-151 | CP000453 |
| Contig370 | 768 | Porifera | *Callyspongia sp.* | 1 | 0 | JN242195 |
| Contig371 | 681 | Porifera | *Ciocalypta sp.* | 1 | 0 | JQ034562 |
| Contig372 | 491 | Gammaproteobacteria | *Vibrio splendidus* | 0 | 2.00E-110 | FM954973 |
| Contig373 | 676 | Mollusca | *Galeomma turtoni* | 0 | 1.00E-119 | AF120658 |
| Contig374 | 691 | Alphaproteobacteria | *Candidatus Pelagibacter ubique HTCC1062* | 0 | 0 | CP000084 |
| Contig375 | 696 | Porifera | *Monanchora arbuscula* | 1 | 0 | EF519645 |
| Contig376 | 706 | Cnidaria | *Calycella syringa* | 0 | 0 | AY789916 |
| Contig377 | 680 | Porifera | *Halichondriid sp.* | 1 | 0 | AY561985 |
| Contig378 | 795 | Porifera | *Tethya coccinea* | 1 | 0 | JQ034584 |
| Contig379 | 709 | Porifera | *Callyspongia fallax* | 1 | 0 | JN242193 |
| Contig380 | 698 | Cnidaria | *Savalia savaglia* | 0 | 0 | DQ825686 |
| Contig381 | 698 | Porifera | *Cliona chilensis* | 1 | 0 | HM999018 |
| Contig382 | 710 | Porifera | *Axechina raspailioides* | 1 | 0 | JQ034546 |
| Contig383 | 707 | Porifera | *Neopetrosia seriata* | 1 | 0 | JN242213 |
| Contig384 | 712 | Porifera | *Cliona celata* | 1 | 0 | HM999035 |
| Contig385 | 711 | Porifera | *Amorphinopsis fenestrata* | 1 | 0 | JQ034540 |
| Contig386 | 686 | Porifera | *Xestospongia muta* | 1 | 0 | EU237490 |
| Contig387 | 622 | Annelida | *Crucigera sp.* | 0 | 2.00E-073 | HM473344 |
| Contig388 | 699 | Mollusca | *Galeomma turtoni* | 0 | 2.00E-148 | AF120658 |
| Contig389 | 798 | Alphaproteobacteria | *Dinoroseobacter shibae DFL 12* | 0 | 1.00E-115 | CP000830 |
| Contig390 | 696 | Porifera | *Oceanapia sp.* | 1 | 0 | JN242223 |
| Contig391 | 685 | Porifera | *Rhizaxinella sp.* | 1 | 0 | AY561983 |
| Contig392 | 699 | Annelida | *Chloeia parva* | 0 | 2.00E-142 | EU352320 |
| Contig393 | 705 | Porifera | *Xestospongia muta* | 1 | 0 | EU237490 |
| Contig394 | 732 | Alphaproteobacteria | *Mesorhizobium ciceri biovar biserrulae WSM1271* | 0 | 1.00E-081 | CP002447 |
| Contig395 | 714 | Arthropoda | *Solenopsis invicta* | 0 | 0 | HQ215540 |
| Contig396 | 695 | Porifera | *Desmapsamma anchorata* | 1 | 0 | HE591461 |
| Contig397 | 682 | Chordata | *Homo sapiens* | 0 | 0 | AP008436 |
| Contig398 | 700 | Porifera | *Iotrochota birotulata* | 1 | 0 | EU237486 |
| Contig399 | 715 | Porifera | *Iotrochota birotulata* | 1 | 0 | EU237486 |
| Contig400 | 697 | Porifera | *Plocamionida sp.* | 1 | 0 | FR687230 |
| Contig401 | 708 | Porifera | *Iotrochota birotulata* | 1 | 0 | EU237486 |
| Contig402 | 715 | Porifera | *Plocamionida sp.* | 1 | 0 | FR687230 |
| Contig403 | 587 | Cnidaria | *Cordylophora sp.* | 0 | 1.00E-024 | EF540793 |
| Contig404 | 712 | Porifera | *Monanchora arbuscula* | 1 | 0 | EF519645 |
| Contig405 | 679 | Gammaproteobacteria | *Pseudoalteromonas haloplanktis* | 0 | 0 | CR954246 |
| Contig406 | 683 | Alphaproteobacteria | *Candidatus Puniceispirillum marinum IMCC1322* | 0 | 8.00E-129 | CP001751 |
| Contig430 | 693 | Annelida | *Chloeia parva* | 0 | 1.00E-113 | EU352320 |
| Contig431 | 1166 | Chordata | *Pyura praeputialis* | 0 | 3.00E-054 | FJ528622 |
| Contig432 | 674 | Chordata | *Didemnum candidum* | 0 | 0 | AY116602 |
| Contig433 | 694 | Annelida | *Chloeia parva* | 0 | 3.00E-102 | EU352320 |
| Contig407 | 518 | Porifera | *Artemisina melana* | 1 | 3.00E-095 | EF519575 |
| Contig408 | 684 | Porifera | *Tethya actinia* | 1 | 0 | AY320033 |
| Contig409 | 675 | Porifera | *Xestospongia muta* | 1 | 0 | EU237490 |
| Contig410 | 691 | Porifera | *Microciona prolifera* | 1 | 0 | DQ087475 |
| Contig411 | 678 | Porifera | *Xestospongia muta* | 1 | 0 | EU237490 |
| Contig412 | 690 | Gammaproteobacteria | *Alkalilimnicola ehrlichii MLHE-1* | 0 | 3.00E-127 | CP000453 |
| Contig413 | 700 | uncultured organism | *environmental samples uncultured organism* | 0 | 1.00E-081 | FJ541275 |
| Contig414 | 687 | Porifera | *Tethya actinia* | 1 | 0 | AY320033 |
| Contig415 | 716 | Porifera | *Cymbastela vespertina* | 1 | 0 | JQ034539 |
| Contig416 | 745 | Porifera | *Mycale fibrexilis* | 1 | 0 | AJ843890 |
| Contig417 | 682 | Porifera | *Thrinacophora cervicornis* | 1 | 0 | JQ034585 |
| Contig418 | 684 | Porifera | *Hymeniacidon sinapium* | 1 | 0 | EF217331 |
| Contig419 | 707 | Porifera | *Phakettia euctimena* | 1 | 0 | JQ034578 |
| Contig420 | 681 | Porifera | *Xestospongia muta* | 1 | 0 | EU237490 |
| Contig421 | 681 | Porifera | *Rhizaxinella sp.* | 1 | 0 | AY561983 |
| Contig422 | 699 | Porifera | *Phakettia euctimena* | 1 | 0 | JQ034578 |
| Contig423 | 687 | Annelida | *Pectinaria granulata* | 0 | 1.00E-144 | DQ209258 |
| Contig424 | 683 | Porifera | *Halichondriid sp.* | 1 | 0 | AY561985 |
| Contig425 | 698 | Porifera | *Callyspongia fallax* | 1 | 0 | JN242193 |
| Contig426 | 693 | Porifera | *Halichondria okadai* | 1 | 0 | EF217340 |
| Contig427 | 706 | Porifera | *Haliclona sp.* | 1 | 0 | JN242210 |
| Contig428 | 695 | Porifera | *Xestospongia muta* | 1 | 0 | EU237490 |
| Contig429 | 699 | Annelida | *Chloeia parva* | 0 | 7.00E-142 | EU352320 |
| Contig434 | 732 | Annelida | *Crucigera sp.* | 0 | 1.00E-114 | HM473344 |
| Contig435 | 689 | Cnidaria | *Parazoanthus tunicans* | 0 | 0 | AB247353 |
| Contig436 | 678 | Porifera | *Acantheurypon pilosella* | 1 | 0 | JF440337 |
| Contig437 | 726 | Porifera | *Neofibularia hartmani* | 1 | 0 | JF773145 |
| Contig438 | 712 | Alphaproteobacteria | *Rhodopseudomonas palustris* | 0 | 7.00E-136 | CP000250 |
| Contig519 | 598 | Porifera | *Clathria schoenus* | 1 | 0 | EF519607 |
| Contig524 | 605 | Porifera | *Clathria schoenus* | 1 | 0 | EF519607 |
| Contig525 | 595 | Porifera | *Thrinacophora cervicornis* | 1 | 0 | JQ034585 |
| Contig439 | 707 | Porifera | *Neofibularia hartmani* | 1 | 0 | JF773145 |
| Contig440 | 710 | Porifera | *Microciona prolifera* | 1 | 0 | DQ087475 |
| Contig441 | 687 | uncultured marine organism | *environmental samples uncultured marine organism* | 0 | 9.00E-147 | GQ234900 |
| Contig442 | 710 | Porifera | *Clathria schoenus* | 1 | 0 | EF519607 |
| Contig443 | 698 | Porifera | *Neofibularia hartmani* | 1 | 0 | JF773145 |
| Contig444 | 696 | Annelida | *Hediste atoka* | 0 | 1.00E-163 | AB603870 |
| Contig445 | 702 | Porifera | *Mycale laxissima* | 1 | 0 | EF519649 |
| Contig446 | 697 | Porifera | *Negombata magnifica* | 1 | 0 | AM420314 |
| Contig447 | 195 | Annelida | *Crucigera sp.* | 0 | 5.00E-029 | HM473344 |
| Contig448 | 730 | Porifera | *Neofibularia hartmani* | 1 | 0 | JF773145 |
| Contig449 | 698 | Porifera | *Iotrochota birotulata* | 1 | 0 | EU237486 |
| Contig450 | 676 | Annelida | *Crucigera sp.* | 0 | 3.00E-128 | HM473344 |
| Contig451 | 696 | uncultured marine organism | *environmental samples uncultured marine organism* | 0 | 1.00E-144 | GQ234900 |
| Contig452 | 669 | Alphaproteobacteria | *Rhodobacter sphaeroides ATCC 17025* | 0 | 4.00E-138 | CP000661 |
| Contig453 | 723 | Gammaproteobacteria | *Colwellia psychrerythraea* | 0 | 4.00E-057 | CP000083 |
| Contig454 | 844 | Gammaproteobacteria | *Xanthomonas axonopodis pv. citri str. 306* | 0 | 2.00E-062 | AE008923 |
| Contig455 | 684 | Chordata | *Didemnum candidum* | 0 | 2.00E-160 | AY116602 |
| Contig456 | 709 | Porifera | *Mycale laxissima* | 1 | 0 | EF519649 |
| Contig457 | 687 | Sipuncula | *Phascolosoma esculenta* | 0 | 1.00E-125 | EF521190 |
| Contig458 | 685 | Porifera | *Echinodictyum mesenterinum* | 1 | 0 | JQ034557 |
| Contig459 | 527 | Alphaproteobacteria | *Ruegeria pomeroyi DSS-3* | 0 | 2.00E-020 | CP000031 |
| Contig460 | 757 | Alphaproteobacteria | *Paracoccus denitrificans* | 0 | 2.00E-130 | CP000489 |
| Contig461 | 757 | Porifera | *Neofibularia hartmani* | 1 | 0 | JF773145 |
| Contig462 | 777 | Porifera | *Diacarnus spinipoculum* | 1 | 0 | AY561975 |
| Contig463 | 693 | Alphaproteobacteria | *Rhodobacter sphaeroides ATCC 17025* | 0 | 4.00E-158 | CP000661 |
| Contig464 | 711 | Cnidaria | *Savalia savaglia* | 0 | 0 | DQ825686 |
| Contig465 | 737 | Alphaproteobacteria | *Dinoroseobacter shibae DFL 12* | 0 | 4.00E-145 | CP000830 |
| Contig466 | 739 | Alphaproteobacteria | *Nitrobacter hamburgensis* | 0 | 1.00E-051 | CP000319 |
| Contig520 | 576 | Annelida | *Crucigera sp.* | 0 | 3.00E-152 | HM473344 |
| Contig521 | 732 | Alphaproteobacteria | *Dinoroseobacter shibae DFL 12* | 0 | 7.00E-155 | CP000830 |
| Contig522 | 715 | Alphaproteobacteria | *Dinoroseobacter shibae DFL 12* | 0 | 9.00E-141 | CP000830 |
| Contig523 | 736 | Alphaproteobacteria | *Dinoroseobacter shibae DFL 12* | 0 | 6.00E-150 | CP000830 |
| Contig526 | 699 | Gammaproteobacteria | *Methylomonas methanica* | 0 | 1.00E-125 | CP002738 |
| Contig527 | 715 | Porifera | *Echinodictyum cancellatum* | 1 | 0 | JQ034555 |
| Contig528 | 509 | Cnidaria | *Parazoanthus sp.* | 0 | 0 | EU591620 |
| Contig529 | 709 | Porifera | *Negombata magnifica* | 1 | 0 | AM420314 |
| Contig530 | 719 | Annelida | *Crucigera sp.* | 0 | 1.00E-127 | HM473344 |
| Contig531 | 729 | Alphaproteobacteria | *Dinoroseobacter shibae DFL 12* | 0 | 3.00E-146 | CP000830 |
| Contig532 | 723 | Annelida | *Crucigera sp.* | 0 | 0 | HM473344 |
| Contig533 | 628 | Porifera | *Artemisina melana* | 1 | 0 | EF519575 |
| Contig534 | 700 | Sipuncula | *Phascolosoma esculenta* | 0 | 3.00E-128 | EF521190 |
| Contig535 | 703 | Porifera | *Neofibularia hartmani* | 1 | 7.00E-180 | JF773145 |
| Contig467 | 718 | Alphaproteobacteria | *Azospirillum brasilense* | 0 | 7.00E-098 | HE577327 |
| Contig468 | 704 | Porifera | *Rhizaxinella sp.* | 1 | 0 | AY561983 |
| Contig469 | 659 | Alphaproteobacteria | *Polymorphum gilvum SL003B-26A1* | 0 | 3.00E-171 | CP002568 |
| Contig470 | 697 | Porifera | *Tethya seychellensis* | 1 | 0 | EF584569 |
| Contig471 | 690 | Porifera | *Hymeniacidon sp.* | 1 | 0 | JQ034565 |
| Contig472 | 692 | Alphaproteobacteria | *Candidatus Puniceispirillum marinum IMCC1322* | 0 | 2.00E-053 | CP001751 |
| Contig473 | 633 | Betaproteobacteria | *Azoarcus sp.* | 0 | 5.00E-067 | AP012304 |
| Contig474 | 687 | Alphaproteobacteria | *Azospirillum brasilense* | 0 | 2.00E-098 | HE577327 |
| Contig475 | 671 | Porifera | *Cliona chilensis* | 1 | 0 | HM999018 |
| Contig476 | 686 | Porifera | *Pione velans* | 1 | 0 | AY561981 |
| Contig477 | 637 | Porifera | *Cliona chilensis* | 1 | 0 | HM999020 |
| Contig478 | 752 | Porifera | *Axinella corrugata* | 1 | 0 | AY791693 |
| Contig479 | 538 | Porifera | *Cymbastela coralliophila* | 1 | 2.00E-071 | JQ034538 |
| Contig481 | 711 | Alphaproteobacteria | *Azospirillum lipoferum* | 0 | 7.00E-092 | FQ311868 |
| Contig482 | 706 | Alphaproteobacteria | *Nitrobacter hamburgensis* | 0 | 6.00E-099 | CP000319 |
| Contig484 | 700 | Gammaproteobacteria | *Alkalilimnicola ehrlichii MLHE-1* | 0 | 9.00E-160 | CP000453 |
| Contig485 | 721 | Alphaproteobacteria | *Azospirillum lipoferum* | 0 | 4.00E-069 | FQ311868 |
| Contig504 | 724 | Alphaproteobacteria | *Azospirillum brasilense* | 0 | 2.00E-112 | HE577327 |
| Contig505 | 707 | Porifera | *Ianthella basta* | 1 | 0 | JF915539 |
| Contig506 | 730 | PX clade | *Cystophora platylobium* | 0 | 5.00E-049 | GU289279 |
| Contig507 | 708 | Porifera | *Pseudoceratina sp.* | 1 | 0 | EF043378 |
| Contig508 | 686 | Porifera | *Ianthella basta* | 1 | 0 | JF915539 |
| Contig509 | 685 | Porifera | *Ianthella basta* | 1 | 0 | JF915542 |
| Contig510 | 706 | Porifera | *Pseudoceratina sp.* | 1 | 0 | EF043378 |
| Contig511 | 740 | Porifera | *Ianthella basta* | 1 | 0 | JF915539 |
| Contig512 | 742 | Alphaproteobacteria | *Azospirillum brasilense* | 0 | 2.00E-111 | HE577327 |
| Contig513 | 715 | Porifera | *Ianthella basta* | 1 | 0 | JF915542 |
| Contig514 | 727 | Porifera | *Ianthella basta* | 1 | 0 | JF915542 |
| Contig515 | 695 | Porifera | *Ianthella basta* | 1 | 0 | JF915539 |
| Contig516 | 699 | Porifera | *Ianthella basta* | 1 | 0 | JF915539 |
| Contig517 | 743 | Porifera | *Axinella corrugata* | 1 | 0 | AY791693 |
| Contig518 | 707 | Porifera | *Ianthella basta* | 1 | 0 | JF915542 |
| Contig536 | 732 | Chordata | *Didemnum candidum* | 0 | 1.00E-133 | AY116602 |
| Contig537 | 670 | Alphaproteobacteria | *Dinoroseobacter shibae DFL 12* | 0 | 9.00E-141 | CP000830 |
| Contig538 | 738 | Alphaproteobacteria | *Ruegeria pomeroyi DSS-3* | 0 | 1.00E-151 | CP000031 |
| Contig539 | 769 | Porifera | *Clathria schoenus* | 1 | 5.00E-138 | EF519607 |
| Contig540 | 710 | Porifera | *Iotrochota birotulata* | 1 | 0 | AY561963 |
| Contig541 | 647 | Annelida | *Crucigera sp.* | 0 | 3.00E-147 | HM473344 |
| Contig542 | 725 | Alphaproteobacteria | *Rhodobacter sphaeroides ATCC 17025* | 0 | 2.00E-155 | CP000661 |
| Contig543 | 705 | Porifera | *Microciona prolifera* | 1 | 0 | DQ087475 |
| Contig544 | 732 | Alphaproteobacteria | *Dinoroseobacter shibae DFL 12* | 0 | 6.00E-156 | CP000830 |
| Contig545 | 715 | Alphaproteobacteria | *Rhodobacter sphaeroides ATCC 17025* | 0 | 6.00E-156 | CP000661 |
| Contig546 | 628 | Alphaproteobacteria | *Rhodobacter sphaeroides ATCC 17025* | 0 | 2.00E-130 | CP000661 |
| Contig547 | 680 | Alphaproteobacteria | *Rhodobacter sphaeroides ATCC 17025* | 0 | 7.00E-142 | CP000661 |
| Contig548 | 704 | Mollusca | *Galeomma turtoni* | 0 | 6.00E-105 | AF120658 |
| Contig549 | 712 | Annelida | *Syllis monilata* | 0 | 2.00E-130 | EF123781 |
| Contig550 | 828 | Porifera | *Diacarnus spinipoculum* | 1 | 0 | AY561975 |
| Contig551 | 772 | Alphaproteobacteria | *Dinoroseobacter shibae DFL 12* | 0 | 1.00E-081 | CP000830 |
| Contig552 | 668 | Porifera | *Microciona prolifera* | 1 | 0 | DQ087475 |
| Contig553 | 698 | Porifera | *Microciona prolifera* | 1 | 0 | DQ087475 |
| Contig554 | 688 | Cnidaria | *Bonneviella sp.* | 0 | 8.00E-116 | AY789893 |
| Contig555 | 707 | Porifera | *Echinodictyum cancellatum* | 1 | 0 | JQ034555 |
| Contig556 | 684 | Porifera | *Echinodictyum cancellatum* | 1 | 0 | JQ034555 |
| Contig557 | 701 | Porifera | *Microciona prolifera* | 1 | 0 | AJ704978 |
| Contig558 | 701 | Porifera | *Strongylacidon bermudae* | 1 | 4.00E-095 | AJ843889 |
| Contig559 | 686 | uncultured marine organism | *environmental samples uncultured marine organism* | 0 | 2.00E-162 | GQ234900 |
| Contig560 | 745 | Porifera | *Neofibularia hartmani* | 1 | 0 | JF773145 |
| Contig561 | 728 | Alphaproteobacteria | *Rhodobacter sphaeroides ATCC 17025* | 0 | 4.00E-152 | CP000661 |
| Contig562 | 829 | Porifera | *Diacarnus spinipoculum* | 1 | 0 | AY561975 |
| Contig563 | 666 | Porifera | *Axinella infundibuliformis* | 1 | 0 | HQ379410 |
| Contig565 | 698 | Annelida | *Crucigera sp.* | 0 | 0 | HM473344 |
| Contig566 | 1245 | Alphaproteobacteria | *Ruegeria pomeroyi DSS-3* | 0 | 1.00E-091 | CP000031 |
| Contig567 | 746 | Porifera | *Neofibularia hartmani* | 1 | 0 | JF773145 |
| Contig568 | 631 | Porifera | *Chelonaplysilla erecta* | 1 | 0 | EF519582 |
| Contig569 | 683 | Alphaproteobacteria | *Rhodobacter sphaeroides ATCC 17029* | 0 | 9.00E-103 | CP000577 |
| Contig570 | 717 | Porifera | *Igernella notabilis* | 1 | 0 | EU237485 |
| Contig571 | 720 | Porifera | *Igernella notabilis* | 1 | 0 | EU237485 |
| Contig572 | 641 | Arthropoda | *Eurythenes gryllus* | 0 | 1.00E-081 | AY830436 |
| Contig573 | 670 | Gastrotricha | *Tetranchyroderma sp.3* | 0 | 7.00E-117 | JF432035 |
| Contig574 | 683 | Porifera | *Halichondria okadai* | 1 | 0 | EF217340 |
| Contig575 | 699 | Porifera | *Verongula gigantea* | 1 | 0 | AM076984 |
| Contig576 | 699 | Porifera | *Chelonaplysilla erecta* | 1 | 0 | EF519582 |
| Contig577 | 688 | Betaproteobacteria | *Azoarcus sp.* | 0 | 7.00E-142 | AM406670 |
| Contig578 | 692 | Arthropoda | *Eurythenes gryllus* | 0 | 6.00E-086 | AY830440 |
| Contig579 | 709 | Porifera | *Igernella notabilis* | 1 | 0 | EU237485 |
| Contig580 | 705 | Porifera | *Verongula gigantea* | 1 | 0 | AM076984 |
| Contig581 | 699 | Porifera | *Igernella notabilis* | 1 | 0 | EU237485 |
| Contig582 | 602 | Porifera | *Plocamionida sp.* | 1 | 0 | FR687230 |
| Contig583 | 730 | Alphaproteobacteria | *Rhodobacter sphaeroides ATCC 17025* | 0 | 8.00E-148 | CP000661 |
| Contig584 | 708 | Porifera | *Halichondriid sp.* | 1 | 0 | AY561985 |
| Contig585 | 646 | Annelida | *Chloeia parva* | 0 | 2.00E-124 | EU352320 |
| Contig586 | 676 | Porifera | *Cinachyrella schulzei* | 1 | 0 | HM032746 |
| Contig587 | 719 | Porifera | *Cinachyrella schulzei* | 1 | 0 | HM032745 |
| Contig588 | 628 | Porifera | *Hemiasterella sp.* | 1 | 0 | AY561977 |
| Contig589 | 709 | Porifera | *Rhabdastrella globostellata* | 1 | 0 | HM592683 |
| Contig590 | 698 | Porifera | *Neofibularia hartmani* | 1 | 0 | JF773145 |
| Contig591 | 696 | Porifera | *Cinachyrella schulzei* | 1 | 0 | HM032745 |
| Contig592 | 713 | Porifera | *Cinachyrella schulzei* | 1 | 0 | HM032745 |
| Contig593 | 707 | Porifera | *Cinachyrella schulzei* | 1 | 0 | HM032745 |
